# Supplementary material for: A Highly Sensitive and Flexible Capacitive Pressure Sensor Based on a Porous Three-Dimensional PDMS/Microsphere Composite
Source: Polymers (Basel). 2020 Jun 24;12(6):1412. doi: 10.3390/polym12061412 (PMC7361688; doi:10.3390/polym12061412)
Supplement: Supplementary file 1 [file polymers-12-01412-s001.pdf]

Supporting Information

A Highly Sensitive and Flexible Capacitive Pressure Sensor Based on a Porous Three-Dimensional PDMS/Microsphere Composite

*Young Jung[1,2], Wookjin Lee[1], Kyungkuk Jung[2,3], Byunggeon Park[1,2], Jinhyoung Park[1], Jongsoo Ko[2], Hanchul Cho[1,]**

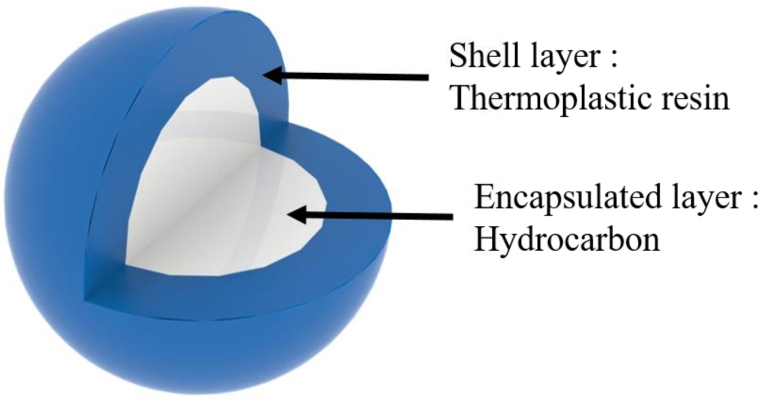

Figure S1. The structure of the microsphere. The microsphere consists of thermoplastic resin and hydrocarbon.

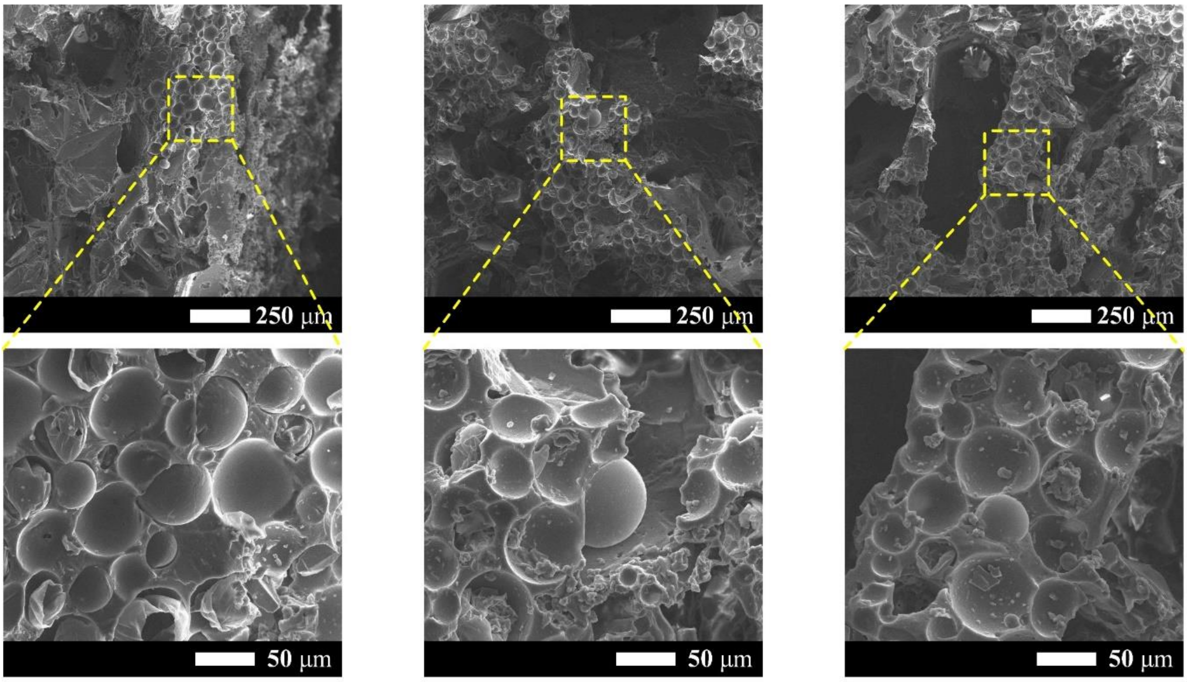

Figure S2. SEM images of the various parts of the PDMS/MS composite. The MS are densely arrayed onto the PDMS structure. Some of the MS disappeared or popped because of the cutting force applied to the device during cutting.

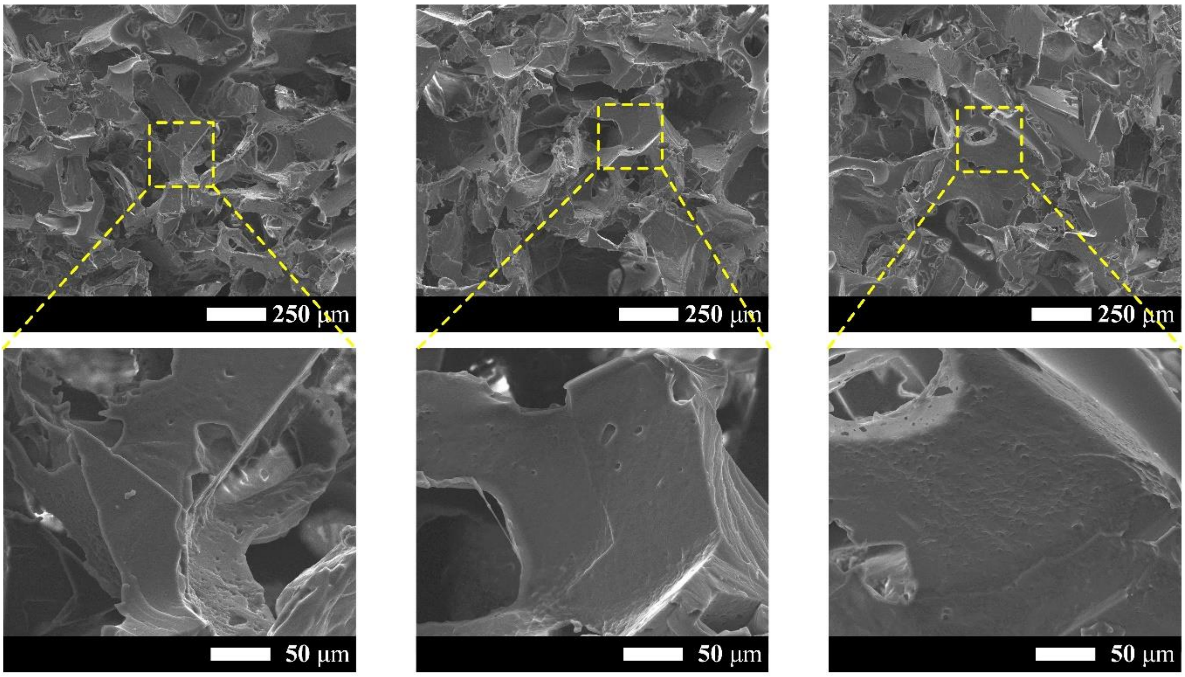

Figure S3. SEM images of the porous PDMS structure. The porous PDMS structure only consists of the PDMS support structure with smooth surface

Figure S4. Porosity calculation results of the porous PDMS structure and PDMS/MS composite. The volume of the porous structure and bulk was equal to 16 x 16 x 10 cm^3.

$$P = \left(\frac{D_s}{D_b}\right) = \frac{(\frac{W}{V})_s}{(\frac{W}{V})_b} = \frac{W_s}{W_b} \tag{S1}$$

Where, D_s is the density of the porous structure, D_b is the density of bulk, W_s is the weight, and V_s is the volume of the porous structure. W_b is the weight, and V_b is the volume of the bulk, respectively.

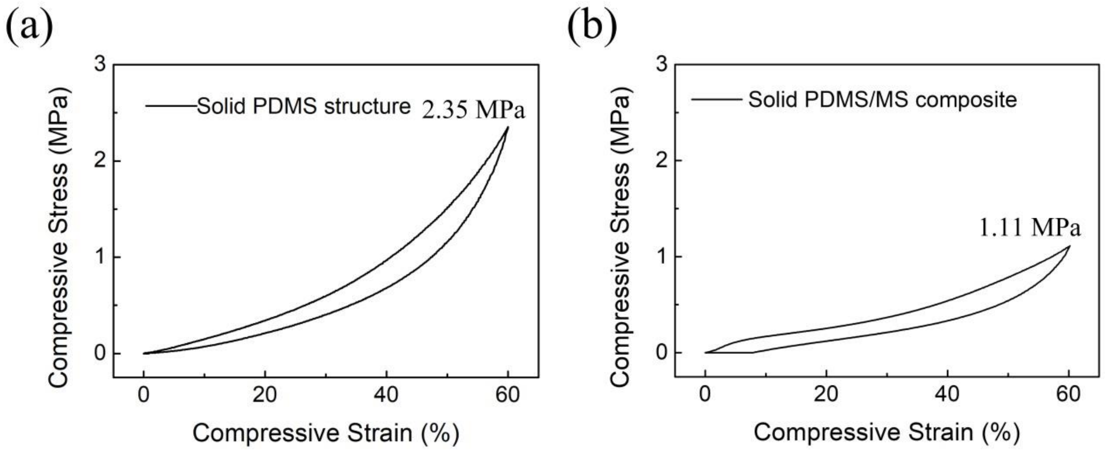

Figure S5. The measured compressive stress-strain curves of the bare solid PDMS structure and PDMS/MS composite for FE simulations. (a) Solid PDMS structure. (b) Solid PDMS/MS composite. Solid PDMS/MS composite is more easily compressed than solid PDMS structure when the microsphere is added.

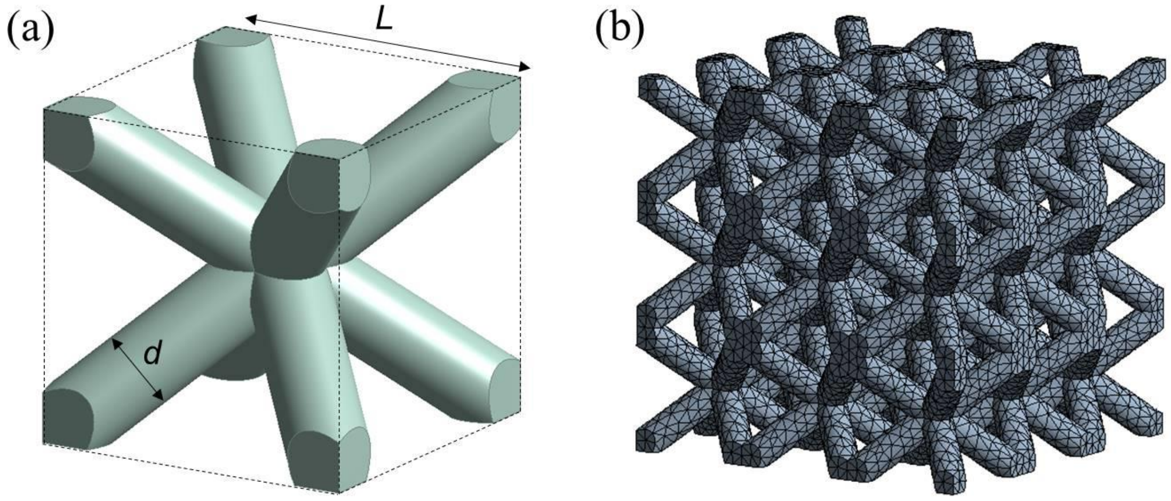

Figure S6. Detailed porous lattice unit cell structure used in FE simulations. We varied the *d/L* of the unit cell, where *d* is the diameter of the strut and *L* is the length of the cube unit cell

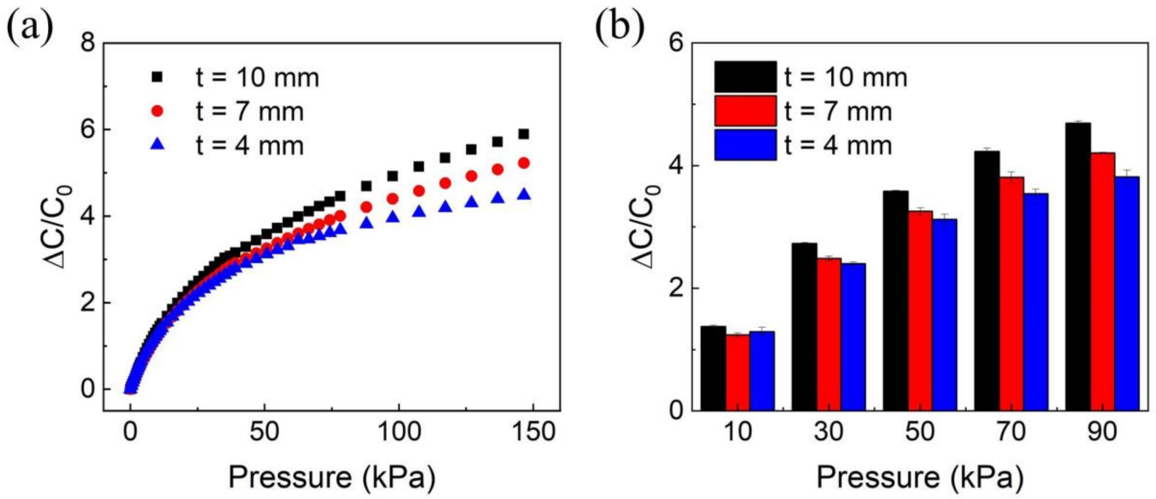

Figure S7. Sensing performance results for pressure sensors of different thicknesses. (a) Measurement capacitance variation with different thicknesses. (b) Comparison of capacitance values at applied pressure.

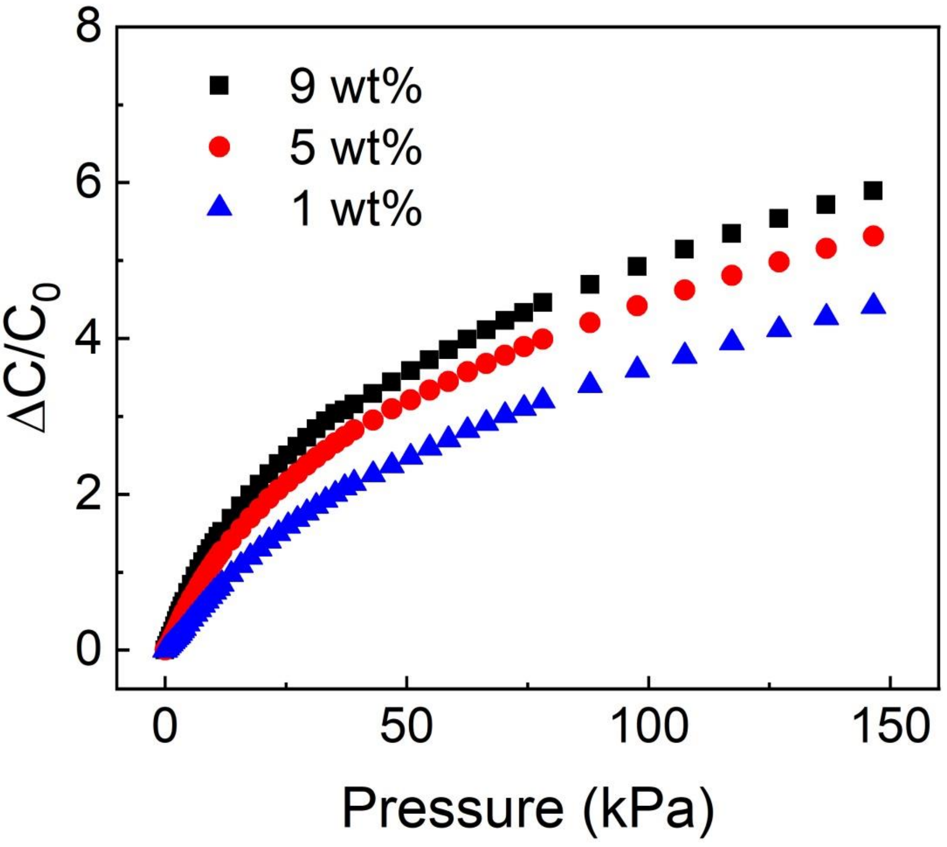

Figure S8. Sensing performance results for pressure sensors of MS concentration.

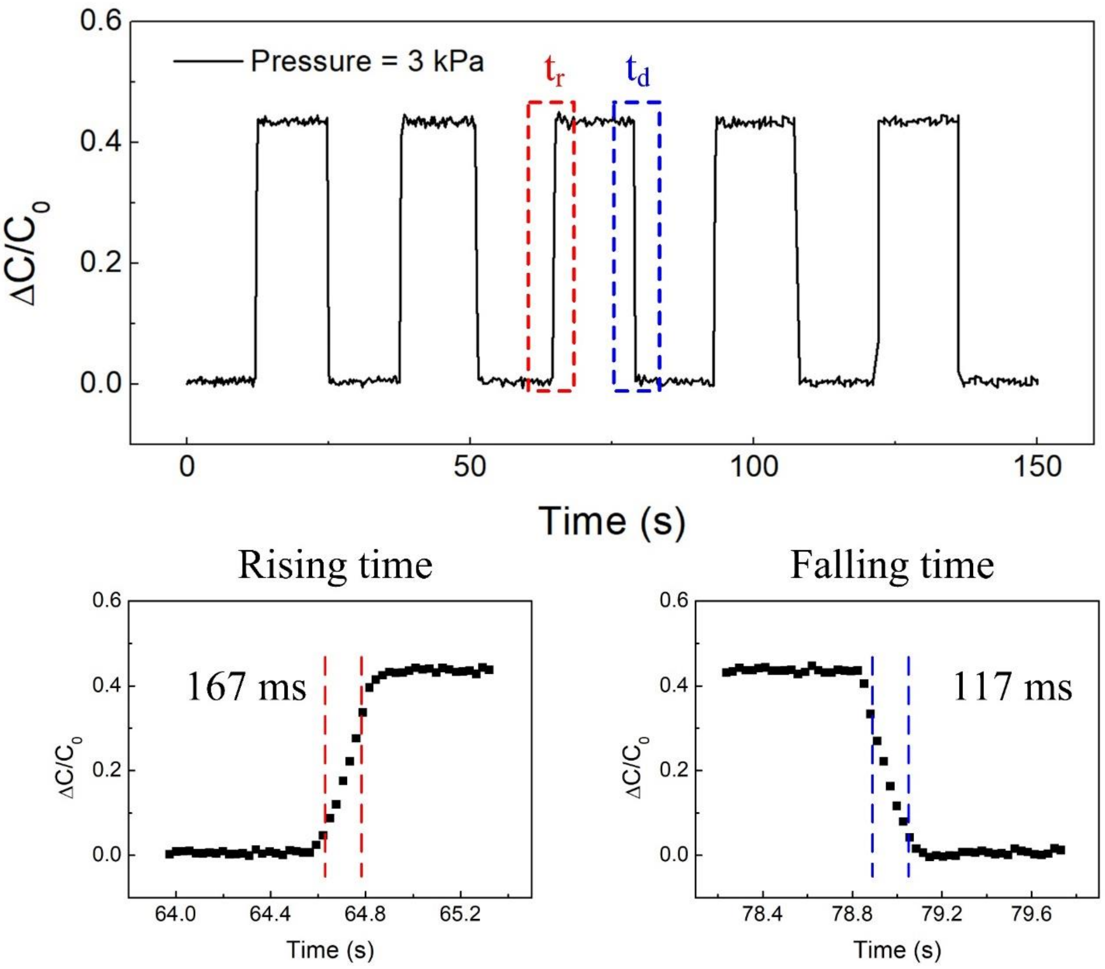

Figure S9. Response time of PDMS/MS sensor for loading 3 kPa. The 10% to 90% rise time (t_r) and the 90% to 10% fall time (t_d) are measured as 167 ms and 117 ms, respectively.

Table S1. Material properties of the porous PDMS structure and PDMS/MS composite with different MS weights. Properties investigated include density, porosity, compressive stress at 60% compressive strain, and Young's modulus at 10% compressive strain (linear section).

	PDMS structure	PDMS/MS composite		
		1 wt%	5 wt%	9 wt%
Density (g/cm^3)	0.277	0.219	0.211	0.195
Porosity (%)	65.3	72.7	73.6	75.6
Compressive stress (kPa)	42.98	32.6	23.4	14.06
Young's modulus (kPa)	2.27	1.80	1.37	0.73

Table S2. Summarization of the reports on capacitive pressure sensor based on porous PDMS structure with nanoparticles.

Materials and structure	Sensitivity (kPa^{-1})	Pressure range	Response time (ms)	Reliability	ref
Calcium copper titanate / PDMS	1.66 (0~640 Pa)	1.4 kPa	<33	None	[1]
Silver nanoparticles / PDMS	0.0072	100 kPa	None	5,000 times / 50, 100 kPa	[2]
Carbon nanoparticles / PDMS	1.1 (0~10 kPa)	100 kPa	< 60	11,000 times / 0.4 kPa	[3]
Microsphere / PDMS	0.124 (0~15 kPa)	150 kPa	< 117	1,000 times / 2.5 kPa	This work

1. Mu, C.; Li, J.; Song, Y.; Huang, W.; Ran, A.; Deng, K.; Huang, J.; Xie, W.; Sun, R.; Zhang, H. Enhanced Piezocapacitive Effect in CaCu3Ti4O12-Polydimethylsiloxane Composited Sponge for Ultrasensitive Flexible Capacitive Sensor. ACS Appl. Nano Mater. 2018, 1, 274–283, doi:10.1021/acsanm.7b00144.

2. Liu, S.Y.; Lu, J.G.; Shieh, H.P.D. Influence of Permittivity on the Sensitivity of Porous Elastomer-Based Capacitive Pressure Sensors. IEEE Sens. J. 2018, 18, 1870–1876, doi:10.1109/JSEN.2017.2789242.

3. Peiqi Wei, Xiaoliang Guo, Xianbo Qiu, D.Y. Flexible capacitive pressure sensor with sensitivity and linear measuring range enhanced based on porous composite of carbon conductive paste and polydimethylsiloxane. Nanotechnology 2019, 30.

Theoretical analysis of capacitance

The capacitance of the parallel-plate capacitor is related by

$$C = \varepsilon_0 \varepsilon_r \frac{A}{d} \tag{S2}$$

Where, ε_0 is the vacuum permittivity, ε_r is the relative permittivity, A is the area of the electrode, and d is the distance between the two parallel sensing electrodes. The relative change in capacitance is calculated as follows:

$$\frac{\triangle C}{C_0} = \frac{C - C_0}{C_0} = \frac{C}{C_0} - 1 \tag{S3}$$

Under the applied pressure, the distance of two electrodes and relative permittivity are changed as the compressive distance (d_c) and effective relative permittivity (ε'). Substituting the initial and compression state capacitance, we get

$$\frac{\triangle C}{C_0} = \frac{\varepsilon_0 \varepsilon_r' \frac{A}{d_c}}{\varepsilon_0 \varepsilon_r \frac{A}{d_0}} - 1 = \frac{d_0}{d_c} \frac{\varepsilon_r'}{\varepsilon_r} - 1 \tag{S4}$$

We expressed the relative permittivity as a function of initial vacuum fraction (porosity) of the PDMS structure f_0, the relative dielectric constant of air ε_a, and the relative permittivity of the polymer ε_p.

$$\varepsilon_r = f_0 \times \varepsilon_a + (1 - f_0) \times \varepsilon_p, \varepsilon_r' = f \times \varepsilon_a + (1 - f) \times \varepsilon_p \tag{S5}$$

Thus, relative change in capacitance is calculated as follows:

$$\frac{\triangle C}{C_0} = \frac{d_0}{d_c} \left\{ \frac{f \times \varepsilon_a + (1-f) \times \varepsilon_p}{f_0 \times \varepsilon_a + (1-f_0) \times \varepsilon_p} \right\} - 1 \tag{S6}$$

We assumed the vacuum fraction f' decreases linearly with the compressive strain ϵ,

$$f = f_0 - a\epsilon \tag{S7}$$

Where, a is the arbitrary constant, ϵ is the strain.

$$\frac{\triangle C}{C_0} = \frac{d_0}{d_c}\left\{\frac{(f_0 - a\epsilon)\times\varepsilon_a + (1 - f_0 + a\epsilon)\times\varepsilon_p}{f_0\times\varepsilon_a + (1 - f_0)\times\varepsilon_p}\right\} - 1 \tag{S8}$$

Simplifying the equation, we get

$$\frac{\triangle C}{C_0} = \frac{d_0}{d_c}\left\{\frac{(f_0\times\varepsilon_a + (1 - f_0)\times\varepsilon_p}{f_0\times\varepsilon_a + (1 - f_0)\times\varepsilon_p} + \frac{a\epsilon\varepsilon_p - a\epsilon\varepsilon_a}{f_0\times\varepsilon_a + (1 - f_0)\times\varepsilon_p}\right\} - 1 \tag{S9}$$

Substituting the values $\varepsilon_a = 1$, $\varepsilon_p = 3$, in Eq S9, we get

$$\frac{\triangle C}{C_0} = \frac{d_0}{d_c}\left(1 + \frac{3a\epsilon - a\epsilon}{f_0\times 3 - 3f_0}\right) - 1 \tag{S10}$$

The relative change in capacitance is then given by the equation:

$$\frac{\triangle C}{C_0} = \frac{d_0}{d_c}\left(1 - \frac{2a\epsilon}{2f_0 - 3}\right) - 1 \tag{S11}$$

Analytical calculation of capacitance

We assume the vacuum fraction f decreases linearly with the compressive strain ϵ.

$$f = f_0 - a\epsilon \quad \text{(S12)}$$

If we assume that the porous structure is fully compressed at the 60% strain (compressed air fraction is zero), the equation can be written as,

$$0 = f_0 - a \times 0.6 \quad \text{(S13)}$$

The porosity of the PDMS structure and PDMS/MS composite is 65.3% (0.653) and 75.6% (0.756), respectively. The arbitrary constants of the PDMS structure and PDMS/MS composite are 1.088 and 1.26, respectively. If the porosity and arbitrary constant are substituted in the equation S10, the equation becomes a function that is determined only by the strain. The strain increased by 2% for a 0 to 60% strain range.
